# Considerations for Secondary Prevention of Nutritional Deficiencies in High-Risk Groups in High-Income Countries

**DOI:** 10.3390/nu10010047

**Published:** 2018-01-05

**Authors:** Maaike J. Bruins, Julia K. Bird, Claude P. Aebischer, Manfred Eggersdorfer

**Affiliations:** DSM Nutritional Products, Wurmisweg 576, Kaiseraugst CH-4303, Switzerland; julia.bird@DSM.com (J.K.B.); claude-p.aebischer@DSM.com (C.P.A.); manfred.eggersdorfer@DSM.com (M.E.)

**Keywords:** nutrient inadequacies and deficiencies, nutritional supplements, biomarkers, nutrition screening, public health, cost-effectiveness

## Abstract

Surveys in high-income countries show that inadequacies and deficiencies can be common for some nutrients, particularly in vulnerable subgroups of the population. Inadequate intakes, high requirements for rapid growth and development, or age- or disease-related impairments in nutrient intake, digestion, absorption, or increased nutrient losses can lead to micronutrient deficiencies. The consequent subclinical conditions are difficult to recognize if not screened for and often go unnoticed. Nutrient deficiencies can be persistent despite primary nutrition interventions that are aimed at improving dietary intakes. Secondary prevention that targets groups at high risk of inadequacy or deficiency, such as in the primary care setting, can be a useful complementary approach to address persistent nutritional gaps. However, this strategy is often underestimated and overlooked as potentially cost-effective means to prevent future health care costs and to improve the health and quality of life of individuals. In this paper, the authors discuss key appraisal criteria to consider when evaluating the benefits and disadvantages of a secondary prevention of nutrient deficiencies through screening.

## 1. Introduction

Primary prevention in the nutrition setting aims to control risk factors in the general population, such as the dissemination of dietary recommendations to improve nutritional knowledge and enable behavior change [1]. There is a widespread use of primary public health strategies, such as the development and promotion of consumer-based dietary guidelines to improve overall dietary quality in many countries [2,3,4]. Despite this, survey data in high-income economies show a moderate burden of nutrient deficiencies and dietary inadequacies for several vitamins and minerals, both in vulnerable population groups and in the overall population [5,6,7,8]. A complementary secondary prevention strategy attempts to identify individuals with nutrient deficiencies, with a focus on high-risk population groups [1]. Secondary prevention detects individuals at risk of disease through screening and other forms of risk appraisal [1]. Secondary prevention should always complement existing programs that are aimed at improving public health along the continuum of disease risk from the well population to managed chronic disease [1]. 

The objective of this paper is to discuss key appraisal criteria to consider when evaluating the benefits and disadvantages of a secondary prevention of nutrient deficiencies through screening.

## 2. Nutrient Inadequacies and Deficiencies

There are a number of interacting factors that can contribute to marginal or low nutrient status, including poor dietary quantity or quality, increased requirements, increased metabolic losses, or impaired gastrointestinal digestion or absorption [9]. The long-term consumption of poor dietary quantity (e.g., due to loss of appetite) or quality (e.g., restrictive, unbalanced, or low-nutrient dense diets [10,11]) increase the risk of poor nutritional status, particularly in individuals with increased needs or losses. Meeting the daily nutrient requirements from the diet is particularly challenging at certain life cycle stages; during pregnancy and lactation [12], infancy and childhood [13], and adolescence [14], nutritional needs for rapid growth and development are significantly increased. In older age groups, many changes, including physical, physiological, and psychosocial factors make it more difficult for nutritional needs to be met, leading to shortfalls in nutrients [15]. In critically ill patients with injury or infectious disease, hypermetabolism is often seen, which is associated with losses and low status of nitrogen, vitamins, and minerals [16,17]. Other factors that contribute to the increased risk for nutritional deficiencies include impaired nutrient absorption capacity (e.g., in gastrointestinal disorders, such as inflammatory bowel disease or coeliac disease, or impaired vitamin B12 absorption in the elderly [18]), poor nutrient bioavailability (e.g., low absorption of iron and zinc from plant-based diets [19]), low bioconversion (e.g., low bioconversion of provitamin A carotenoids from plant-based diets into vitamin A [20]). Other factors that can increase the risk of specific nutrient deficiencies include for instance the use of some medication [21] or genetic polymorphisms [22]. 

The initial stages of marginal nutrient deficiency are often overlooked, as they may remain asymptomatic for a long time or present with generalized signs and symptoms that may not be recognized by the health care professional [23]. When unrecognized, subclinical symptoms can progress into more severe clinical deficiency states [24,25]. For example, vitamin B12 and folate deficiency can both present as megaloblastic anemia, symptoms of which include weakness and fatigue, neurological effects, such as numbness and tingling in the hands and feet, and poor memory. Vitamin B12 deficiency is a particular problem in older adults who are less able to absorb the vitamin, as well as vegetarians and vegans who consume little vitamin B12 from animal foods [25]. Low folate status in pregnant women is rated as a risk factor for neural tube defects in offspring and poor pregnancy outcomes [25], yet blood levels that are required to prevent neural tube defects are much higher than needed to prevent folate deficiency [26]. Iron deficiency anemia can lead to tiredness, weakness, a weakened immune system, and impaired memory [24,25]. Iron deficiency anemia is common in women with heavy menstrual bleeding, pregnant women, infants and young children, vegetarians and vegans, and people with gastrointestinal disorders. A low vitamin D status has little outward signs initially, but leads to bone pain, muscle weakness, and eventually increased fracture rates if left untreated [24,25]. Vitamin D deficiency is prevalent worldwide, and risk groups include older adults, postmenopausal women, people with dark skin, breastfed infants, and people with gastrointestinal malabsorption conditions [25]. 

## 3. Public Health Problem of Inadequate Intakes and Deficiencies of Nutrients

Surveys show that, even in high-income countries, nutrient intakes fail to meet requirements for many people, and overall nutrient status is too low for several essential nutrients [27]. Particularly in vulnerable population groups, specific nutrient inadequacies and deficiencies can present a public health issue [27]. Nutrient deficiencies not only have short-term implications for health and quality of life, but also, long-term consequences for intellectual development and economic productivity [28]. Nevertheless, relatively few efforts have been undertaken in high-income countries to estimate the potential public health benefits and cost savings of overcoming nutrient deficiencies of public health concern. 

**Figure 1:**
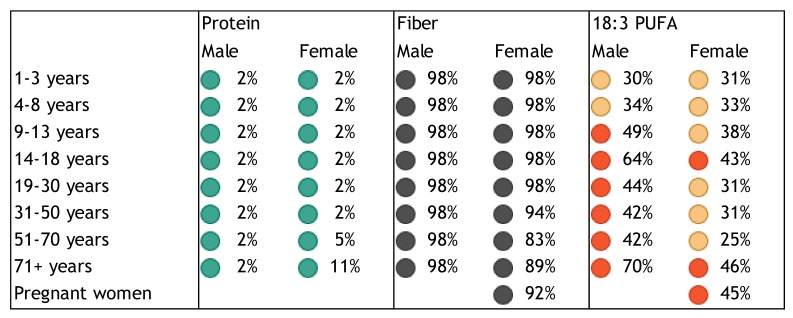
Proportion of inadequate macronutrient intakes by age, gender and life stage categories based on percentage of the US population with intakes below the Estimated Average Requirement (EAR) (protein) or adequate intake (fiber, 18:3 PUFA). From National Health and Nutrition Examination Survey (NHANES) 2007–2010). Inadequate intakes: black: >80%, red: 40–80%, yellow: 20–40%, and green: <20% below EAR or adequate intake.

**Figure 2:**
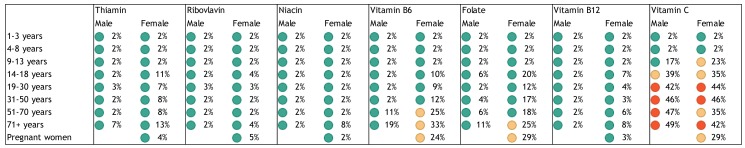
Proportion of inadequate intakes of water-soluble vitamins by age, gender and life stage categories based on percentage of the US population with intakes below the EAR. From NHANES 2007–2010). Inadequate intakes: black: >80%, red: 40–80%, yellow: 20–40%, and green: <20% below EAR.

**Figure 3:**
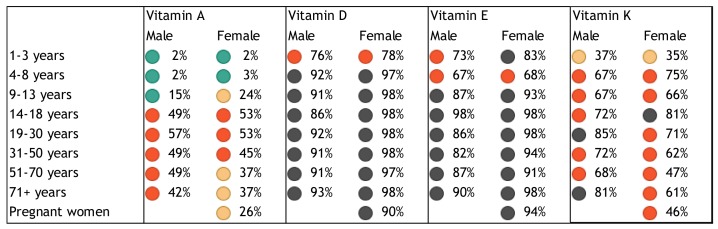
Proportion of inadequate intakes of fat-soluble vitamins by age, gender and life stage categories based on percentage of the US population with intakes below the EAR (vitamins A, D, and E) or adequate intake (vitamin K). From NHANES 2007–2010). Inadequate intakes: black: >80%, red: 40–80%, yellow: 20–40%, and green: <20% below EAR or adequate intake.

**Figure 4:**
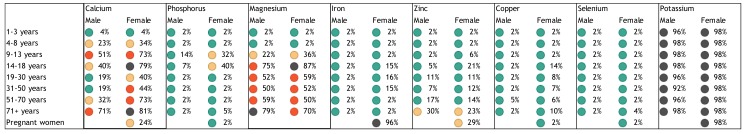
Proportion of inadequate intakes of minerals by age, gender and life stage categories based on percentage of the US population with intakes below the EAR (calcium, phosphorus, magnesium, iron, zinc, copper, selenium) or adequate intake (potassium). From NHANES 2007–2010). Inadequate intakes: black: >80%, red: 40–80%, yellow: 20–40%, and green: <20% below EAR or adequate intake.

**Figure 5:**
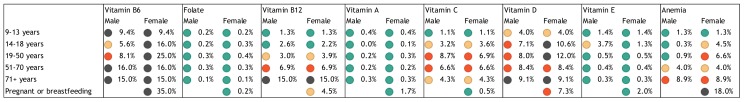
Risk of deficiency by age, gender and life stage categories for individual vitamins or anemia, based on percentage of the population aged >9 years with biomarkers below the deficiency cut-off values (vitamin B6, folate, B12, A, C, D, E, and anemia; pyridoxal 5′-phosphate <20 nmol/L; serum folate <2 ng/mL or red blood cell folate <95 ng/mL; vitamin B12 <200 pg/mL or methylmalonic acid >0.271 µmol/L; serum retinol <20 µg/dL; vitamin C <0.2 mg/dL; 25-hydroxyvitamin D <12 ng/mL; α-Tocopherol <500 µg/dL; mean corpuscular volume <95 fL, respectively). Deficient status: black: >9%, red: 6–9%, yellow: 3–6%, and green: <3% below the cut-off value for deficiency. Based on NHANES data 2003–2006.

Population groups at particular risk of nutrient deficiencies include women of childbearing age, especially pregnant and lactating women [29,30,31,32,33], infants and toddlers [34,35], children [33,36], adolescents [31], older adults [33,34,37], obese individuals [38], and the critically ill [17]. Based on representative data from the National Health and Nutrition Examination Survey (NHANES), the US Office of Disease Prevention and Health Promotion classified vitamins A, C, D, E, and folate, calcium, and magnesium as “nutrients of concern” that may pose a substantial public health concern in the general US population [29]. The risk of single or multiple, concurrent micronutrient deficiencies in children and adults based on NHANES data was recently estimated at 31% [33]. We present a quantitative assessment of the burden of poor nutritional intake and status in Figure 1, Figure 2, Figure 3, Figure 4 and Figure 5 to illustrate the risk of deficiency in a high-income country. Figure 1, Figure 2, Figure 3 and Figure 4 show the proportion of inadequate nutrient intakes in the US population for macronutrients, water-soluble vitamins, fat-soluble vitamins, and minerals, respectively, as estimated using a Usual Intake distribution calculated according to the National Cancer Institute Method [39]. Figure 5 shows biochemical nutrient deficiencies in the US population, as calculated using the NHANES 2003–2006 dataset. The method and ethics approval are described in [33]. Briefly, the proportion individuals not meeting established cut-points for deficiency for the US population aged nine years or more was calculated, taking the complex sample design into account, and weighted to be representative. Figure 1, Figure 2, Figure 3 and Figure 4 show that vitamins A, folate, B9, C, D, E, K, magnesium, calcium, potassium, fiber, and long chain omega-3 polyunsaturated fatty acids, are under-consumed compared to the Estimated Average Requirement (EAR). Figure 5 shows a prevalence of over 6% for anemia and vitamin B6, B12, C, and D deficiency, in several sub-population groups for each micronutrient. 

In other high-income regions, surveys demonstrate that certain macronutrient, vitamin, and mineral deficiencies can be prevalent. For instance, in Arabian Gulf countries, despite year-long sunshine, vitamin D deficiency remains a critical health concern increasing from childhood through adolescence [40,41]. The spectrum of micronutrient deficiencies in Europe and Central Asia and their public health consequences have recently been published by the Food and Agriculture Organization of the United Nations (FAO) [42]. The data show that even in high-income countries like Germany, Austria, France, and UK, iron deficiency anemia (>10%) and zinc deficiency (>4%) are still highly prevalent, and the related disease burden in terms of Disability-Adjusted Life Years (DALYs) is substantial, responsible for 268 DALYs per 100,000 population in the whole Europe and Central Asia region. The recent Global Burden of Disease study demonstrates that unbalanced diets (both over- and under-consumption) contribute considerably to the global disease burden [42,43]. Lack of dietary fiber, seafood long-chain omega-3 polyunsaturated fatty acids, and calcium are among the leading food and nutrient risk factors contributing to the global burden of disease in high-income economies [43].

## 4. Common Approaches to Prevent Nutrient Inadequacies and Deficiencies

Policy makers are increasingly aware of the public burden and associated costs of under- and over-nutrition. This has resulted in various public health strategies to improve lifestyles and dietary choices, and prevent nutrient deficiencies considered of public health concern. Primary prevention measures taken to ensure more appropriate nutrient intakes in the population often include (1) education programs to encourage healthier and more nutritious food choices; (2) food-based approaches that increase the availability or affordability of nutrient-rich foods; and, (3) national policies to fortify commonly-eaten foods [44]. 

Encouraging appropriate intakes of healthy nutrient-rich and balanced diets is generally the preferred strategy for meeting nutrient needs, if possible. This can be effective in improving nutrient status, whilst improving overall dietary quality. A recent review of diets quality, demonstrated that increasing unhealthy patterns are outpacing increases in healthy patterns in most world regions particularly in high-income countries [45]. These findings emphasize the continued need for primary prevention strategies to address suboptimal diet quality, both by encouraging consumption of nutritious foods and discouraging consumption of unhealthy foods. 

Nutrition surveys in high-income countries have identified that some of the shortfall nutrients continue to persist (see paragraph 3). To address the problem of persistent nutrient inadequacies and deficiencies, policy makers can consider a “secondary prevention strategy” complementary to a population-based primary prevention approach. Secondary prevention involves selecting population subgroups at risk of nutrient deficiencies, in order to administer additional nutritional support to those at greatest need, for example, dietetic services, dietary supplementation, or another approach. Secondary prevention programs, like the US Women’s Special Supplemental Food Program for Women, Infants, and Children (WIC), which attempt to address nutrition-related problems in multiple subgroups of the population at risk using a multiple-service integrated food- or education-based approach can be more complex to implement [46]. Another more targeted secondary prevention approach seeks to prevent specific nutrient deficiencies in subgroups at high risk, through screening and targeted intervention at first point of contact with the health care professional.

In most high-income health care economies, health care expenditure is largely directed towards inpatient and outpatient care, and medical goods (mainly pharmaceuticals). For instance, in the US, only 6% is spent on public health and prevention services [47]. Despite evidence that preventive strategies in general practice, such as lifestyle interventions and screening for diabetes could have a large impact on population health, they remain underutilized in Australia [48]. The US Centers for Disease Control and Prevention (CDC) also emphasize that chronic disease conditions are often less expensive to treat when they are detected early and still preventable, recommending that the population should have access to affordable preventative services [49]. The CDC have developed a Diabetes Prevention Impact Toolkit to help employers, insurers, and health departments to calculate the costs and benefits of national diet and physical activity change programs [49]. For secondary nutrition and dietary preventions strategies, the benefits in terms of higher quality of life, less hospitalization, health care costs, and increased productivity are less-well investigated.

Access to dietitian services in primary care for those at risk of diet-related nutrient inadequacies is largely underutilized in most countries [50]. Yet, in an increasing number of countries, preventative health checks are offered as secondary prevention to identify individuals at risk of disease. For instance, screening for cardiovascular risk factors, such as dyslipidemia, have been effective in reducing the overall burden of cardiovascular disease [51]. Screening tests for nutrient inadequacies and deficiencies in asymptomatic individuals are generally not covered by insurance plans. However, some tests are becoming more common as part of an annual health check-up or routine testing in risk groups (e.g., iron deficiency anemia in pregnant women, vitamin D in people at risk, or vitamin B12 status in older adults [52,53,54,55,56]). Nevertheless, nutrient deficiencies are often still recognized and are treated in an unnecessarily late stage, despite the availability of biomarkers allowing the early detection and management of nutritional inadequacies and deficiencies before the onset of symptoms. More focus on implementing evidence-based nutrition strategies complementary to public health approaches would prevent unnecessary nutrient inadequacies and deficiencies in vulnerable groups.

## 5. Criteria Determining Cost-Effectiveness

A health economic assessment is necessary to judge whether dietary advice and managing nutrient deficiencies in high-risk groups can be cost-effective, and to come to possible recommendations. Few cost-effectiveness assessments have been performed in the national context. at-risk group. Whether providing nutrition services, including dietary advice and if indicated supplemental nutrients, to groups at risk in the primary care setting can be cost-effective depends on multiple factors, as outlined below. 

### 5.1. Public Health and Economic Consequences

First, the public health and economic consequences of nutrient inadequacies and deficiencies are the primary consideration for developing health care policy. The prevalence of nutrient inadequacies or low status is indicative of the public health problem, but the severity of clinical health consequences determines the actual burden of disease. In short, a cost-utility analysis using DALY or Quality Adjusted Life Year (QALY) as an outcome measure is recommended [57]. The analysis should be performed within a timeframe that is long enough to capture the period when the main health effects and costs arise. Sensitivity analysis is also recommended to assess the influence of central assumptions and uncertainty. Preventing nutrient deficiencies in the early years, from conception to five years of age, can be expected to have important effects on lifelong health, physical and mental performance, quality of life, and work capacity, and it is important to consider intergenerational effects [58,59].

### 5.2. Evidence Base Supporting Improved Health Outcomes, Discomfort and Risks

Second, in order to be cost-effective, provision of dietary services or dietary supplements to at-risk groups should lead to the expected improved health outcomes. The compromised health consequences from essential micro- and macro-nutrient inadequacies and deficiencies are generally well described (see paragraph 2). Although the reversal of nutrient deficiencies through re-supplementation can be expected to exert health benefits for the deficient individual, high-quality evidence from randomized controlled trials is not always available. The baseline nutrient status of the population and dose-response effects of nutrient re-supplementation should be considered where available. Effects of nutrient interventions in several high-quality studies can be inconsistent [60]. Unexplained inconsistency of nutritional effects in several high-quality studies may suggest underlying interactions that are yet unknown. Responsiveness to nutrients may be determined by possible gene-nutrient [60,61], nutrient-nutrient [62], or nutrient-drug [63,64] interactions. Guideline development for nutrition recommendation is often driven by requirements for high-quality evidence from randomized controlled intervention trials, even though the adverse health consequences of their deficiencies are well-known. An example of high-quality evidence approved by the European Food Safety Authorities is based on a several meta-analyses that suggest the beneficial effect of daily vitamin D supplementation in combination with calcium on the reduced the risk of falling [65].

Dietary supplements (tablets, capsules, liquids, powders) may be recommended in conjunction with a dietary advice if the needs for specific nutrients are difficult to be met by a food-based approach alone. No risks are associated with dietary modification. Dietary supplements are expected to be safe when taken under supervision of a health care professional, and under the conditions recommended, i.e. not exceeding the daily safe upper intake level (UL). A UL has been established for several of the vitamins and minerals by various regulatory bodies, below which intakes are likely to pose no risk of adverse effects. 

For some of the nutrients, the health consequences of long-term high intakes in subgroups of the population remain debated. For instance, there is a large body of literature demonstrating the efficacy of maternal folic acid intake in preventing birth defects [66]. Nevertheless, some findings in literature link very low and very high folic acid intakes to increased cancer risk [66]. Two authoritative bodies that recently evaluated the possible risks from high folic acid intakes, concluded that evidence for adverse effects of high folic acid intake was not conclusive, but recommended further research to identify whether subgroups (e.g., with preexisting neoplasia or specific genetics) might be at an increased risk [66]. Beta-carotene became the subject of controversy when two studies reported that high β-carotene intake for several years was associated with higher risk of lung cancer in smokers [67,68], particularly in those who were lacking glutathione-S transferase 1 and 2 due to genetic variation [67]. Nevertheless, other large studies did not show such an effect [69]. 

Examples of adverse nutrient-drug and nutrient-nutrient interactions include high calcium intake that may adversely affect absorption and efficacy of certain antibiotics [70] or the absorption of dietary iron [71]. Examples of beneficial nutrient-nutrient interactions include the enhancing effect of vitamin C intake on iron absorption [62]. As long as these positive or negative effects of nutrients in special groups of the population are not well-established, their formal incorporation into a cost-benefit assessment remains difficult. 

In rare cases, for instance, when efficacy of oral supplements is limited by malabsorption or intolerance, nutrients can be administrated by intravenous administration. Iron doses that are recommended for the prevention of iron deficiency may cause gastrointestinal when exceeding the UL, particularly when poorly absorbable iron forms are given [72]. Intravenous iron infusion is reserved for severe anemia or in the case of intolerance or unresponsiveness to oral therapy [73]. For vitamin B12, an initial intramuscular injection followed by oral supplements can be recommended if absorption is poor [74]. However, the associated higher costs of intravenous infusion or intramuscular injection might limit widespread use of these administration forms [75]. 

Inconveniences and discomfort involved in biomarker-based screening for deficiencies need to be considered. Blood drawing by venipuncture may cause local pain, bruising, and in rare cases, infection. Minimal or non-invasive methods using finger prick, urine- and saliva-based biomarkers that can be performed directly by the individuals may increase acceptance. The benefits of preventing nutrient deficiency-related clinical symptoms are generally expected to prevail over the minor discomforts of screening. 

### 5.3. Availability of an Accurate Test

A prerequisite to screen individuals at-risk for specific nutrient inadequacies or deficiencies is the availability of a suitable test that has sufficient sensitivity and specificity. For many, but not all, vitamins and minerals, blood-, urine-, and saliva-based biomarkers of status exist, requiring minimally invasive sampling [76]. These biomarkers can detect specific nutrient deficiencies in an early stage before symptoms occur. Sensitive methodologies exist that measure omega-3 polyunsaturated fatty acid status requiring blood draw [77], while finger-prick blood tests are available in some countries. For protein status, no single routine and reliable indicator can be recommended at this time. Inexpensive, less accurate, and/or less predictive biomarkers can also be used in an initial screening, and if indicated, followed by more robust accurate and predictive tests to come to a final diagnosis. Examples include an initial hemoglobin test, followed by a serum ferritin test to accurately diagnose iron deficiency anemia [78], or qualitative lateral flow assays for vitamin D3 to test threshold levels [79]. 

Numerous screening tools have been developed to identify elderly or patients at risk of calorie or protein malnutrition. These tools have the disadvantage that they only detect overt signs of general malnutrition in a late stage, while specific nutrient deficiencies may go unnoticed [80]. Biomarkers indicative of general malnutrition that were found to be useful in older adults included BMI, hemoglobin, and total cholesterol [81]. Other possibilities include the use of validated dietary questionnaires to assess the risk of inadequate intake of (specific) nutrients [82]. However, dietary questionnaires are generally not sensitive and time consuming [83].

“Point-of-care” tests complying with regulatory requirements to diagnose and monitor nutrient inadequacies and deficiencies are expected to become increasingly specific and sensitive in the future. Gold standard testing and procedures for diagnosis, age- and gender-specific ranges, and cut-off levels to define deficiency are needed, although consensus is often lacking. Fast response times and low costs improve the likelihood that physicians and patients accept testing and immediate clinical decisions and guidance policies are met. Time and resource constraints by primary health-care professionals are the main barriers to perform nutrition screening and monitoring in general practice [84], and the actual uptake of a test strongly depends on its convenience for patient and physician. Monitoring to assess improvement in nutrient status or intake may be appropriate in patients with symptomatic deficiency, patients with malabsorption, or when poor adherence is suspected. 

### 5.4. Adoption and Adherence

Third, the effectiveness of an intervention in primary health care strongly depends on its awareness and adoption among health care practitioners; i.e., the proportion aware of the nutrition problem, and the proportion of risk populations testing for deficiency, receiving dietary counseling, and being prescribed a nutrient regimen if indicated [85,86]. A small study showed that health care providers usually do not follow the testing recommendations for vitamin B12 deficiency [87]. Furthermore, the proportion of individuals adhering to the prescribed regimen, or, if applicable, willing to pay out-of-pocket non-reimbursed regimens also determines the effectiveness of secondary prevention [85,86]. 

Adherence rates can be expected to be lower for preventative therapies than for treatments. For example, folic acid supplementation of pregnant women can be a cost-effective means to prevent debilitating neural tube defects in infants [88], yet program effectiveness strongly depends on women taking folic acid in the critical peri-conceptual period when the supplements are effective in reducing risk of neural tube defects. Cost of food is a primary determinant of food choice and the higher nutrient-dense foods, which are associated with higher prices may reduce adherence [89]. The costs of various dietary supplement forms are generally low but can result in different adherence rates; tablets and capsules are generally shelf-stable over a longer time, provide a fixed dose, and their convenience is likely to maintain compliance. Powders and liquids may be an option, particularly for children, who may have difficulty swallowing tablets or capsules, but powders need to be mixed with food making them less convenient, and sometimes their taste may reduce compliance. 

### 5.5. Costs and Cost Savings

Finally, the expected total direct and indirect costs and cost-savings of a secondary prevention strategy should be considered. Direct expenses involve the costs of diagnostic testing, costs of a dietitian consult, and costs of a dietary supplement regimen. Costs of screening tests vary widely, but as an example, a vitamin D deficiency test can cost on average $50. Costs of dietary consultation vary globally, but are generally low (e.g., about $100–$200 in the US). Nutritional counselling aimed at overnutrition was shown to be potentially cost-effective in various settings [90,91]. Nutrition strategies aimed at preventing deficiencies have not been assessed for their costs and benefits. Dietary supplements vary in their price; vitamin and mineral supplements can cost as little as a few cents per serving, whereas, for instance, costs of protein, omega-3 long-chain fatty acids, or fiber supplements can range from $0.20 to $1.20.

Potential direct cost savings include reduced medical care expenses and indirect costs savings involve gains in work productivity resulting from overcoming the deficiency-related health problems. 

**Figure 6:**
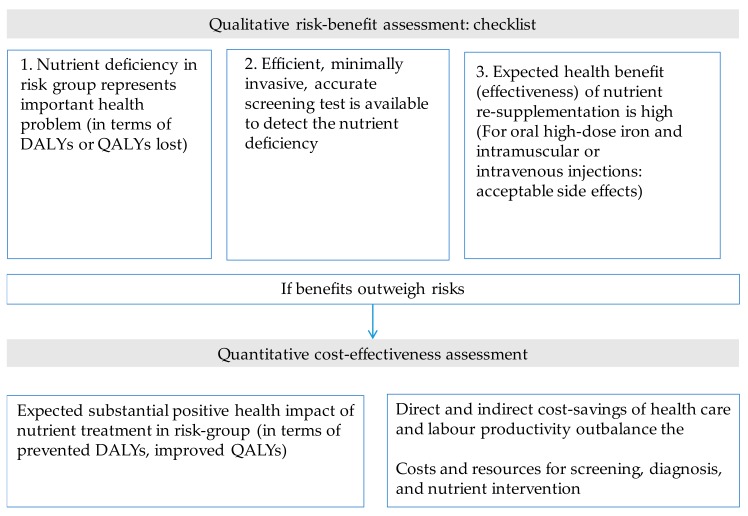
Initial qualitative risk-benefit assessment and subsequent quantitative cost-effectiveness analysis of nutrient supplementation of high-risk groups.

Figure 6 shows a checklist of criteria to be considered when assessing the cost-effectiveness of addressing nutrient inadequacies and deficiencies in a secondary prevention program. In a first step, the benefits of overcoming nutrient deficiencies can be balanced against possible constraints and disadvantages in a qualitative manner. Subsequently, the cost-effectiveness of preventing nutrient inadequacies and deficiencies can be evaluated by quantifying net cost savings and the public health impact. It is recommended that health benefits, disadvantages, and cost-effectiveness be evaluated for different scenarios (e.g., different uptake rates).

### 5.6. Evidence Gaps in Evaluating Secondary Nutrition Strategies

There are several challenges inherent to coming to recommendations for secondary nutrition strategies targeted at subgroups of biggest concern. The main problem is that data needed to come to recommendations of an intended program may be hampered by a lack of certainty, quality, or completeness. 

For example, Rukuni et al. [92] systematically analyzed all the risks and benefits of screening and iron treatment of pregnant women in general practice in the UK to reduce iron deficiency anemia. In this review, several major gaps in the evidence were identified in relation to several criteria, for instance, insufficient evidence from high quality randomized controlled trials that early detection is effective in reducing morbidity and mortality, and robust evaluations of the cost-effectiveness of screening programs for iron deficiency anemia.

The effects of uncertainties can be assessed in health-economic modelling. The UK National Institute for Health and Care Excellence (NICE) modelled different scenarios for the UK, when comparing supplementation of populations at risk of vitamin D deficiency (pregnant and breastfeeding women, children aged under five, and over 65 years of age), either supplied universally to all at risk, or preceded by deficiency screening [86]. The outcomes strongly depended on several criteria, all affected by a degree of uncertainty; the prevalence of symptomatic vitamin D deficiency at baseline and after the intervention; adoption of the vitamin D recommendation among health professional and patients; the health outcomes expected in each scenario; the cost of testing for vitamin D deficiency; the cost of vitamin D supplements; and, the costs of treating symptomatic vitamin D deficiency. Under the assumed scenario, the results showed that testing to identify the deficient people is likely to cost more than universal vitamin D supplementation of the entire at-risk population without prior testing. A disadvantage of the latter is the unnecessary exposure of adequate individuals to unnecessary high intakes.

As the results of the cost-effectiveness analysis depends on the reliability of the data that it is based on, it is worthwhile to quantify uncertainties in base assumptions using sensitivity analyses. 

## 6. Discussion

A risk-benefit balance followed by a more thorough cost-effectiveness assessment will allow for well-balanced recommendations for addressing nutrition deficiencies of major public health concern in a secondary prevention strategy. Some of the input variables dealing with uncertainty include the rate of adoption of nutrition guidelines among program implementers, as well as adherence of individuals to prescribed nutritional therapy. Moreover, high-quality evidence for the benefits of reducing nutrient deficiencies in at-risk groups is urgently needed to allow for appropriate cost-effectiveness analysis of screening for nutrient deficiencies. To judge the total evidence supporting cost-effective nutrition interventions can be challenging and requires certain estimates and assumptions to be embedded into the cost-effectiveness assessment. Nevertheless, uncertainty in inputs can be analyzed and should not prevent the implementation of a cost-effectiveness assessment. 

The various inconsistent recommendations for nutrient management developed by different organizations may be a barrier to effective implementation of a secondary nutrition prevention strategy. Governments should drive policy consensus on guidelines. A more profound problem is the limited access to nutrition services in primary health care: time or expertise of primary health care professionals to counsel individuals on nutrition, access to and collaboration with dietitians or nutritionists, continuous monitoring, and evaluation of the individuals that are at risk, all affect the effectiveness of nutrition interventions. Rapid point-of-care tests are increasingly being administered by trained staff and health care professionals in pharmacies, hospitals, and clinics [93]. In the meantime, increasing the awareness among conscious consumers about the potential link between certain nutrient inadequacies and deficiencies and adverse health outcomes has resulted in an increase in the rate of self-testing [94]. Moreover, the emerging use of self-diagnostic tests by consumers [95] suggests that consumers are becoming more active in diagnosing and managing their own health. In the future, personalized nutritional recommendations based on individuals’ genetic testing will further contribute to this. Ultimately, consumer-driven personalized management of nutrient deficiencies based on testing is likely to develop more rapidly than the implementation of targeted prevention strategies via the health care system. 

## 7. Conclusions

National survey data show that adequate nutrient intakes and sufficient status may be difficult to achieve across all age and gender groups. Primary prevention strategies to avoid nutrient deficiencies are often not sufficient in certain subgroups of the population. Screening those at highest risk, followed by targeted nutrition services, is often underestimated and overlooked as a potentially cost-effective intervention to prevent clinical deficiencies. Whether a secondary nutrition prevention approach could be cost-effective or even cost-saving over the medium to long term, depends on various criteria. Some degree of uncertainty is inherent in such health economic evaluation. If a biomarker test is used for screening, the availability of an affordable, predictive, and efficient test of nutrient status in the at-risk population is important in view of the time and resource constraints general practitioners are facing. The success of guidelines to prevent and control nutrient deficiencies in vulnerable population groups strongly depends on the extent that health care professionals are informed, engaged, and implementing them, and individuals adhere to them. To come to recommendations to improve the nutrient supply to those at risk of being deficient requires a balance of the disadvantages and benefits, and a cost-effectiveness assessment.
